# Don’t Forget the Flu – Determining the Etiology of Infective Myositis in a Child: A Case Report

**DOI:** 10.5811/cpcem.2020.12.50405

**Published:** 2021-01-26

**Authors:** Lauren M. Crowley, Richard J. Mazzaccaro, Amy Lewis Dunn, Sarah E. Bauch, Marna Rayl Greenberg

**Affiliations:** * University of South Florida Morsani College of Medicine, Lehigh Valley Campus, Lehigh Valley Health Network, Department of Emergency and Hospital Medicine, Allentown, Pennsylvania; † University of South Florida Morsani College of Medicine, Lehigh Valley Campus, Lehigh Valley Health Network, Department of Pediatrics, Allentown, Pennsylvania; ‡ University of South Florida Morsani College of Medicine, Lehigh Valley Campus, Lehigh Valley Health Network, Department of Emergency and Hospital Medicine, Division of Pediatric Emergency Medicine, Allenton, Pennsylvania

**Keywords:** Infective myositis, influenza A, upper respiratory tract infection, pediatrics, case report

## Abstract

**Introduction:**

Infective myositis is an acute, self-limited condition, rarely occurring in children with recent viral infections. The condition is often overlooked by emergency physicians when endeavoring to exclude other diagnoses included in the differential. Diagnosis of the condition can be difficult when based purely on clinical presentation because it shares symptoms with much more concerning neurological illnesses. A few simple laboratory tests are indicated to reach the correct diagnosis.

**Case Report:**

The following case report describes a three-year-old female diagnosed with a recent upper respiratory tract infection presenting to the emergency department with complaints of fatigue and inability to walk. She was diagnosed with an influenza-like illness three days prior by her pediatrician, the symptoms of which had mostly resolved by the time of presentation.

**Conclusion:**

Muscle weakness and abnormal, uncoordinated gait with an acute upper respiratory tract infection in a child may be cause for concern, prompting unnecessary work-up. Emergency physicians should be aware of the signs and symptoms of influenza-associated infective myositis in children, especially during influenza season.

## INTRODUCTION

Infective myositis is an acute, self-limiting condition that occurs in children with a history of recent viral infection; while there are reports in those with influenza A, it is a significantly more frequent complication in children with influenza B infections.^1^,^2^ This condition affects males more often than females (about 2:1).^2^ Characteristic clinical and laboratory features of infective myositis include reluctance or refusal to walk, with intense pain in the lower extremities (most notably swelling and tenderness in the gastrocnemius or soleus muscles) causing abnormal gait, elevated serum creatine kinase (CK) levels, and positive viral studies.^3^ Normal or decreased white blood cell and platelet counts are indicative of an immune response to the recent viral infection,^3^ which is most commonly diagnosed as an upper respiratory tract infection.^1^

A complication in diagnosing infective myositis is its onset, which begins in the early convalescent phase of illness, about three days after the onset of fever and respiratory symptoms.^1^ Despite its generally good prognosis,^2^ myositis is a commonly missed diagnosis. Early consideration and diagnosis of myositis may help prevent an extensive diagnostic work-up and unnecessary, costly laboratory testing or neuroimaging. We describe a case of a three-year-old female with infective myositis due to influenza A.

## CASE REPORT

A three-year-old female with a past medical history of asthma presented to the emergency department (ED) with fatigue and generalized body aches. The patient complained that her “legs were tired,” and her mother stated that she was unable to walk and could only crawl since waking that morning. She had been diagnosed with an influenza-like illness three days prior by her pediatrician, the symptoms of which had mostly resolved by the time of presentation except for cough and rhinorrhea. She had been afebrile for 36 hours prior to ED presentation.

Vital signs included the following: blood pressure 108/89 millimeters of mercury, temperature 37.6°Celsius, respirations 22 breaths per minute, and oxygen saturation on room air of 99%. Physical examination was remarkable for cervical lymphadenopathy and abnormal coordination and gait. Specifically, the child was alert with mentation appropriate for age, and she had no pupil abnormalities and no slurring of speech. The remainder of her neurological exam (including reflexes) was normal and without focal deficit. The patient had a wide-based, shuffling gait, and while she slightly favored her right leg and hip she had no restricted range of motion of the hips or knees and had no pain in the joints to movement or palpation.

Laboratory serum chemistries were significant for elevated CK levels (319 units per liter (U/L) [normal <201 U/L]) indicating myositis; creatinine 0.34 milligrams per deciliter (mg/dL) (0.39–0.55 mg/dL); alkaline phosphatase 128 U/L (156–369 U/L); and aspartate aminotransferase 65 U/L (26–55 U/L). Trace amounts of protein were found in her urine. Rapid strep A test was negative. Comprehensive respiratory pathogen profile polymerase chain reaction detected influenza A-matrix and influenza A-H3. Complete blood count results showed decreased white blood cell count (3.1 thousand per centimeters cubed [K/cm^3^] [6.0–17.0 K/cm^3^]) consistent with a viral infection. This patient had not been vaccinated for that year’s strain of influenza.

The patient was admitted from the ED for observation overnight and given intravenous fluids, oseltamivir, and ketorolac to treat the flu and pain and inflammation, respectively. Infective myositis of the bilateral lower extremities was determined as the principal problem. By the next morning, she was able to walk without significant pain, showed no signs of ataxia, remained afebrile, and tolerated oral medications. The patient was discharged home 27 hours after initial presentation and instructed to continue ibuprofen every six hours for the next one to two days.

## DISCUSSION

Given that most cases of infective myositis are in males with influenza B, this case of a young female with infective myositis due to influenza A is less common.^2^ To avoid potential neurological damage, physicians quickly assessed her condition for the best outcome. Upon admission, the initial concern was for cerebellar ataxia with flu-like symptoms due to difficulty walking, muscle weakness, and abnormal coordination. In general, it can be difficult to distinguish between pain from muscle inflammation, muscle weakness, and uncoordinated ambulation in young children due to their lack of cooperation and developmental stage.^3^ In a child with myositis, the gait can be nonspecific; it sometimes mimics ataxia but can also appear as “toe-walking” due to pain and resistance to flexing and extending at the ankle.^3^

**CPC-EM Capsule**

What do we already know about this clinical entity?

Infective myositis is an acute, self-limited condition, rarely occurring in children with recent viral infections.

What makes this presentation of disease reportable?

The condition is often overlooked by emergency physicians when trying to exclude other diagnoses included in the differential.

What is the major learning point?

Emergency physicians should be aware of the signs and symptoms of influenza-associated infective myositis in children, especially during influenza season.

How might this improve emergency medicine practice?

Early consideration and diagnosis of myositis may help prevent an extensive diagnostic work-up and unnecessary, costly lab testing or neuroimaging.

Children with myositis do not need the extensive work-up that children with ataxia sometimes need. Because the patient demonstrated normal reflexes, had no speech abnormalities, headaches, seizures, or altered mental status, and had no history of trauma, toxic ingestion, or a previous ataxic episode, diagnoses such as acute cerebellitis, stroke, peripheral neuropathy, and metabolic disorders were able to be excluded.^4^ Imaging and radiology studies were deemed unnecessary in this case, but may be indicated for longer duration of ataxic symptoms or if the patient’s history suggests possibility of intracranial pathology.^5^

The most significant historical detail in this case was the patient’s recent upper respiratory tract infection. Viral studies were ordered to determine the underlying cause of infection. Influenza A is known to be associated with infective myositis as well as conditions such as Guillain-Barré syndrome (GBS), rhabdomyolysis, and acute, post-infectious cerebellar ataxia.^1^,^6^,^7^ Guillain-Barré syndrome is a post-infectious disorder that may cause ataxia. Patients generally experience bilateral, symmetric lower extremity weakness, often with the presence of sensory symptoms, and pain that can present as a refusal to walk in a young, non-verbal child.^6^ Absent or decreased deep-tendon reflexes is a principal feature of GBS, and cerebrospinal fluid protein levels are often elevated as determined by lumbar puncture (LP).^6^ Since these clinical defining criteria were not present, we ruled out GBS in our case and a LP was not performed. Had we not been as confident in the clinical exam, an LP could have been done to look for cytoalbuminologic dissociation.

Rhabdomyolysis is a rare complication of myositis, possibly leading to renal failure or compartment syndrome. Influenza A and B, parainfluenza, coxsackievirus infection, Epstein-Barr virus, herpes simplex, adenovirus, and cytomegalovirus have been associated with rhabdomyolysis.^8^ Influenza A is more common in cases of rhabdomyolysis than influenza B, and more often occurs in young females than young males – the opposite of common characteristics found with infective myositis.^1^,^2^

Acute post-infectious cerebellar ataxia accounts for about 30–50% of acute ataxia cases in children^7^ and is the most common cause of acute ataxia in the post-varicella vaccination era.^9^ It should be highly considered in the differential diagnosis of a child with gait abnormalities. Acute post-infectious cerebellar ataxia can qualify as a neurologic emergency due to rapid onset of symptoms in otherwise healthy individuals with normal mental status.^10^ In contrast to our patient, clinically the presentation may include slurring of speech, abnormal coordination in balance, or uncoordinated motions of the hands or feet. It is often the result of an autoimmune-mediated inflammatory response, triggered by an infection.^11^

Varicella zoster virus was the most commonly reported etiology prior to widespread vaccination in children;^12^ overall varicella incidence decreased by 84.6% from the implementation of one-dose to two-dose recommendation.^13^ According to the 2017 National Immunization Survey, an overwhelming majority of children aged 19–35 months received one or more doses of the varicella vaccine (91.0%).^14^ Enterovirus, Epstein-Barr virus, hepatitis A, herpes simplex, influenza, measles, mumps, and parvovirus B19 have been associated with acute ataxia following acute infection^4^,^12^; therefore, one of these viruses is more likely to be related to a child’s post-infectious ataxic symptoms than varicella.

With the recent uptick of enterovirus D68 outbreaks in children over the past 10 years,^15^ physicians may be specifically aware of enterovirus-associated cases of infective myositis in addition to influenza. Additionally, a consideration is acute flaccid myelitis, which can also present with normal mentation and isolated limb weakness and gait abnormalities. Yet more often than not, it is influenza A or B that is responsible for myositis and its clinical manifestations of an antalgic gait and muscle weakness/fatigue. Treatment with oseltamivir may be necessary for influenza symptoms, but the symptoms of myositis typically resolve within a few days with hydration and supportive care.

Based on the severity of the pediatric patient’s presenting symptoms, emergency physicians should consider a wide array of etiologies with a child presenting with inability or refusal to walk, including infection, trauma, intoxication, accidental ingestion, and hereditary and neurological disorders.^3^ Emergency physicians should be aware of the signs and symptoms of infective myositis due to influenza in order to prevent any unnecessary testing and aid in making an accurate clinical judgment.

## CONCLUSION

A case of muscle weakness and abnormal gait in the setting of an acute upper respiratory tract infection in a child may be cause for concern of a serious neurological diagnosis and prompt unnecessary work-up. Performing a thorough neurological exam and the findings of myositis, especially in the right season, can help streamline the appropriate differential and aid in patient care.

## References

[1] Hu JJ, Kao CL, Lee PI (2004). Clinical features of influenza A and B in children and association with myositis. J Microbiol Immunol Infect.

[2] Szenborn L, Toczek-Kubicka K, Zaryczański J (2018). Benign acute childhood myositis during influenza B outbreak. Adv Exp Med Biol.

[3] Magee H, Goldman RD (2017). Viral myositis in children. Can Fam Physician.

[4] Fogel BL (2012). Childhood cerebellar ataxia. J Child Neurol.

[5] Rudloe T, Prabhu SP, Gorman MP (2015). The yield of neuroimaging in children presenting to the emergency department with acute ataxia in the post-varicella vaccine era. J Child Neurol.

[6] Van Doorn PA, Ruts L, Jacobs BC (2008). Clinical features, pathogenesis, and treatment of Guillain-Barré syndrome. Lancet Neurol.

[7] Rossi A, Martinetti C, Morana G (2016). Neuroimaging of infectious and inflammatory diseases of the pediatric cerebellum and brainstem. Neuroimaging Clin N Am.

[8] Nauss MD, Schmidt EL, Pancioli AM (2009). Viral myositis leading to rhabdomyolysis: a case report and literature review. Am J Emerg Med.

[9] Thakkar K, Maricich SM, Alper G (2016). Acute ataxia in childhood: 11-year experience at a major pediatric neurology referral center. J Child Neurol.

[10] Pedroso JL, Vale TC, Braga-Neto P (2019). Acute cerebellar ataxia: differential diagnosis and clinical approach. Arq Neuropsiquiatr.

[11] Naselli A, Pala G, Cresta F (2014). Acute post-infectious cerebellar ataxia due to co-infection of human herpesvirus-6 and adenovirus mimicking myositis. Ital J Pediatr.

[12] Ryan MM, Engle EC (2003). Acute ataxia in childhood. J Child Neurol.

[13] Lopez AS, Zhang J, Marin M (2016). Epidemiology of varicella during the 2-dose varicella vaccination program — United States, 2005–2014. MMWR Morb Mortal Wkly Rep.

[14] Hill HA, Elam-Evans LD, Yankey D (2017). Vaccination coverage among children aged 19–35 months - United States, 2016. MMWR Morb Mortal Wkly Rep.

[15] Uprety P, Curtis D, Elkan M (2019). Association of enterovirus D68 with acute flaccid myelitis, Philadelphia, Pennsylvania, USA, 2009–2018. Emerg Infect Dis.

